# Comparative Scanning Electron Microscopy (SEM) Evaluation of Advanced Irrigation Activation Techniques for Bioceramic Sealer Removal in Retreatment: An In Vitro Study

**DOI:** 10.7759/cureus.113849

**Published:** 2026-08-03

**Authors:** Somya Kumari, Rishika Raman, Chi Koy Wang, Binoy K Singh, Abhinav K Singh

**Affiliations:** 1 Department of Conservative Dentistry and Endodontics, Buddha Institute of Dental Sciences and Hospital, Patna, IND

**Keywords:** bioceramic, diode laser, irrigation activation, retreatment, ultrasonic

## Abstract

Background: Retreatment cases involving bioceramic (BC) sealers consistently present a major challenge, as residual sealer remnants hinder the efficient cleaning and shaping of the root canals. Removing these sealers is difficult with standard syringe irrigation. To overcome this limitation, irrigant activation techniques have been introduced to help clean otherwise inaccessible areas of the root canal system, particularly the apical third. Therefore, this study evaluated the percentage of canal cleanliness along the coronal, middle, and apical thirds of root canal walls using scanning electron microscopy (SEM) following different irrigant activation techniques: laser diode, sonic activation, and passive ultrasonic activation.

Methodology: Eighty single-canal mandibular premolars with completely formed apices were selected. Primary endodontic treatment was performed using hand and rotary instruments, followed by obturation with gutta-percha and a BC sealer. After a four-month incubation period, retreatment was performed using ProTaper Universal Retreatment Files (Dentsply Maillefer, Tulsa, OK). The specimens were then randomly divided into four equal groups (n = 20) based on the irrigation activation protocol applied: Group I received conventional syringe irrigation; Group II received sonic activation; Group III received passive ultrasonic activation; and Group IV received laser diode activation. Following the activation protocols, the samples were sectioned longitudinally and prepared for SEM evaluation. The percentage of clean canal surface area in each third was quantified using ImageJ software. A one-way ANOVA was performed to compare the groups, followed by the Bonferroni post-hoc test for pairwise comparisons.

Results: The percentage of cleaned canal surface area demonstrated statistically significant differences among all four groups across the apical, middle, and coronal regions (p < 0.0001). Group IV consistently exhibited the highest mean cleanliness values across all anatomical locations, followed sequentially by Group III and Group II, while Group I recorded the lowest values.

Conclusions: Within the limitations of this study, none of the irrigant activation methods was capable of completely removing all root canal filling material. However, laser diode irrigation demonstrated the highest efficacy in removing BC sealer from root canals during retreatment.

## Introduction

Endodontic retreatment is a demanding and time-consuming procedure that carries an increased risk of procedural errors [1]. Endodontic failure often stems from an inadequate apical seal that allows persistent bacteria to survive [2]. Root canal sealers prevent this by filling microscopic voids and entombing remaining microorganisms.

Bioceramic (BC) sealers are highly popular among clinicians due to the presence of calcium phosphate, which enhances their setting properties and initiates a beneficial chemical reaction. This reaction forms a natural, tooth-like crystal structure and bone apatite material that improves the sealer's bond to root dentin while lowering the setting time compared to alternative materials. A major disadvantage of these BC sealers, however, is the difficulty of retrieving them from the root canal space after hardening during retreatment or post-space preparation [3].

Proper elimination of sealer and gutta-percha provides better cleaning of the root canal space by removing necrotic material and bacterial residues [4]. Various techniques are used to remove root canal filling materials during retreatment, including hand files, nickel-titanium (NiTi) rotary files, Gates Glidden burs, ultrasonic instruments, lasers, irrigation agitation systems, and solvents. Shaping files alone cannot completely eliminate root canal filling materials during retreatment [5]. To overcome these limitations and enhance antimicrobial efficacy, newer approaches in irrigants and irrigation techniques have been introduced.

Conventional syringe irrigation operates as a positive-pressure technique. The needle is placed up to 1 mm short of the working length, where fluid pressure pushes the irrigant into the canal. However, it has significant limitations in effectively cleaning complex areas, particularly the apical region and canal isthmuses [6].

Activated irrigation techniques are essential because they generate wall shear stress that helps dislodge residual gutta-percha, debris, biofilm, and tissue remnants from the root canal system, thereby improving overall cleaning efficiency. Various activation methods have been developed to enhance the effectiveness of irrigants, including sonic activation, ultrasonic activation, and laser-based systems, such as the diode laser [7]. Sonic activation operates at lower frequencies (approximately 1-10 kHz or 1,500-6,000 Hz) compared to ultrasonic irrigation. Sonic devices, such as the EndoActivator, generate hydrodynamic activity using polymer-based tips. A limitation of these polymer tips is their radiolucency, making clinical detection difficult if separation occurs inside the canal.

Compared to sonic activation, ultrasonic irrigation produces significantly cleaner root canals due to its higher operating frequency (25-40 kHz) and enhanced hydrodynamic effects [8]. Ultrasonic activation improves cleaning primarily through acoustic microstreaming and cavitation [9]. However, it also carries a potential risk of apical extrusion of the irrigating solution [8].

Laser activation, particularly with diode lasers operating at near-infrared wavelengths (810-1,064 nm, specifically 940 nm and 980 nm), can produce cavitation within the irrigating fluid. Cavitation involves the rapid formation and collapse of vapor bubbles, generating strong shear forces along the dentinal walls [10].

While advanced irrigation activation systems have demonstrated varying success in removing the dentinal smear layer during primary endodontic treatment [11,12], their efficacy in retrieving highly resilient BC sealers during retreatment remains insufficiently explored.

The present study aims to compare the efficacy of different irrigation activation systems for the removal of BC sealer from the root canal space during retreatment. The null hypothesis was that there is no significant difference between these activation techniques in terms of canal cleanliness.

## Materials and methods

Study design

After obtaining approval from the Institutional Ethical Committee of the Buddha Institute of Dental Sciences & Hospital, Patna, India (Ref. no. 76/BIDSH), 80 freshly extracted human mandibular premolars - extracted for orthodontic or periodontal reasons - were selected for the study.

Inclusion and exclusion criteria

Teeth with an intact single root, straight canals, completely developed root apices, and no history of previous endodontic treatment or developmental anomalies were included. Conversely, teeth with immature open root apices, a history of trauma, or crowns/roots fractured or destroyed by extensive caries or trauma were excluded.

Sample size estimation and preparation

The sample size was calculated using G*Power software (version 3.1.9.4, Heinrich-Heine-Universität Düsseldorf, Germany). The effect size (f = 0.39) was derived from the mean reduction rate (%) of the volume of filling material reported in a previous study [13]. A minimum total sample size of 80 specimens was estimated across four groups with an alpha error of 0.05 and 80% power, resulting in an allocation of (n= 20) specimens per group.

Decoronation of the teeth was performed at the cementoenamel junction using a diamond disc and a straight handpiece to standardize the root length at 13 mm. After preoperative assessment, access cavities were prepared using a high-speed handpiece (NSK Dental, Hoffman Estates, IL) and burs, including an Endo Access Bur No. 2 and an Endo-Z Bur (Dentsply Maillefer, Switzerland). The canal orifice was located using a DG16 explorer (Dentsply Maillefer, Switzerland). Initial patency was established with a #10 K-file, and the working length was determined to be 1 mm short of the radiographic apex. A glide path was created using a #10 K-file, followed by a rotary glide file according to the manufacturer's instructions.

Biomechanical preparation of the canal was completed using ProTaper Gold rotary files (Dentsply Maillefer, Switzerland) incrementally from the orifice opener Sx up to file F2 (30/.08 apical preparation) at the working length. All files were operated at 500 rpm with a torque of 2.5 N·m using an electric motor. The canal was irrigated with 2.5 mL of 5.25% sodium hypochlorite (NaOCl) and saline using a 30-gauge side-vented needle (Changzhou Super Dental Technology Co., Ltd., China) placed 1 mm short of the working length before the introduction of each file. Canal patency was verified with a #10 K-file after every instrument change. Before final obturation, the canals received a final flush of 5 mL of 5.25% NaOCl, 5 mL of distilled water, 5 mL of 17% ethylenediaminetetraacetic acid (EDTA), and a final rinse of distilled water before being dried with absorbent paper points.

Root canals were obturated with F2 gutta-percha cones (Dentsply Maillefer, Ballaigues, Switzerland) and Bio-C BC sealer (Angelus, Londrina, PR, Brazil) composed of tricalcium silicate, dicalcium silicate, tricalcium aluminate, calcium oxide, zirconium oxide, silicated glass, and additives. Down-pack was completed using a Fi-P obturation pen (Guilin Woodpecker, Guilin, China) at 180°C with a 40/.04 plugger within 3 mm of the working length. Canal spaces were backfilled using a Fi-G obturation gun (Guilin Woodpecker) and compacted with hand pluggers (Waldent, India). Access cavities were sealed with Cavit-G (3M ESPE, Germany). To achieve complete setting, chemical maturation, and biomineralization of the sealer, obturated specimens were incubated at 37°C and 100% humidity for four months prior to retreatment [14]. This extended aging period ensures full sealer hydration, providing a clinically authentic retreatment model.

Randomization and allocation concealment

Specimens were randomized into four experimental groups using a computer-generated random number sequence. Allocation concealment was performed using sequentially numbered, opaque, sealed envelopes that were opened only at the time of obturation and retreatment procedures by an independent investigator who was not involved in the SEM outcome evaluation. All prepared samples were included in the study without exclusion.

Retreatment protocol

ProTaper Universal Retreatment Files (Dentsply Maillefer, Switzerland) were operated at 500-700 rpm. For the coronal third, the D1 file (30/.09) was used to remove the first 4 mm of obturation material. With light apical pressure, the D2 (25/.08) and D3 (20/.07) files were used sequentially to remove the filling material from the middle and apical thirds. Canals were irrigated with 10 mL of 5.25% NaOCl between files. Retreatment instrumentation was deemed complete only when no visible obturating material remained on the instruments or within the canal, which was confirmed radiographically.

Following retreatment, the samples (n = 80) were randomly assigned to four groups (n = 20 each) based on the irrigation activation protocol:

Group I (syringe irrigation): Canals were irrigated with 5 mL of 5.25% NaOCl using a disposable plastic syringe and a 30-gauge side-vented needle placed 1 mm short of the working length using a gentle push-and-pull motion. This was followed by 5 mL of distilled water, 5 mL of 17% EDTA, and a final flush of 5 mL of distilled water.

Group II (sonic irrigation): Canals were filled with 5 mL of 5.25% NaOCl. A polymer tip (size 30/.04; EndoActivator, Dentsply Sirona, Charlotte, NC) was placed 1 mm short of the working length and operated at 10,000 cpm in a vertical push-and-pull motion. The irrigant was activated for one minute (three cycles of 20 seconds each). The same activation protocol was repeated using 5 mL of distilled water, 5 mL of 17% EDTA, and a final 5 mL flush of distilled water.

Group III (passive ultrasonic irrigation): A Mani U-file tip (size 15; Mani, Inc., Japan) was mounted onto a Woodpecker UDS-J Ultrasonic Scaler (Guilin Woodpecker Medical Instrument Co., Ltd., China) operating at a power setting of 9 (30 kHz). The activation tip was intentionally positioned 2 mm short of the working length. This 2 mm stand-off distance was chosen to allow free, unrestricted oscillation of the rigid metallic tip within the apical canal geometry, preventing instrument-to-wall contact, acoustic dampening, and unintentional dentin gouging while ensuring effective acoustic microstreaming and cavitation throughout the full working length. The irrigation regimen comprised 5 mL of 5.25% NaOCl, 5 mL of distilled water, 5 mL of 17% EDTA, and a final 5 mL rinse of distilled water. Each solution was activated for one minute in three 20-second continuous cycles [14].

Group IV (laser diode irrigation): Canals were filled with 5 mL of 5.25% NaOCl and activated using a 980-nm diode laser (Tejas-D, Intense Medical & Dental System Pvt. Ltd., India) equipped with a 200 µm fiber optic tip. The laser tip was placed 1 mm short of the working length and operated at 2.5 W in a continuous push-pull motion. Activation was performed in four cycles of five seconds each, resting for 20 seconds between cycles to avoid thermal damage. The canal was rinsed with 5 mL of distilled water, followed by 5 mL of 17% EDTA activated under the same parameters, and a final flush of 5 mL of distilled water.

The root samples were then partially grooved externally in a buccolingual direction using a diamond disc on a straight handpiece (Nakanishi Inc., Japan) without penetrating the canal space. Horizontal grooves were placed at 4 mm and 8 mm from the root apex to demarcate the coronal, middle, and apical thirds. The roots were gently split longitudinally using a chisel to expose the canal lumen for evaluation.

Final scanning of specimens and image analysis

The split root fragments were dehydrated in an ascending series of ethyl alcohol over eight hours and allowed to dry overnight at room temperature. For surface characterization, both split root fragments (mesial and distal halves) of each sample were mounted on aluminum stubs using double-sided conductive carbon tape. The specimens were sputter-coated with a thin conductive gold layer of approximately 10-15 nm thickness using a rotary-pumped sputter coater (Quorum Q150R, Quorum Technologies Ltd., Laughton, UK) operating with a gold target under a vacuum pressure of 0.1 mbar at a working current of 20 mA for 60 seconds.

Following sample preparation, specimens were examined under a scanning electron microscope (Hitachi S-4800 Field Emission SEM; Hitachi High-Technologies Corp., Tokyo, Japan) across three defined anatomical levels (coronal, middle, and apical thirds) over a standardized working length of 13 mm. Both split fragments were independently evaluated at each level to capture a complete representation of the root canal lumen without selective sampling bias.

For quantitative evaluation, one representative field per root third (coronal, middle, and apical) showing the maximum presence of remaining debris and sealer remnants was photographed at ×10,000 magnification at an accelerating voltage of 10 kV, with a standardized micron scale bar (5 µm) included on each micrograph. To ensure examiner blinding and eliminate observer bias, all digital scanning electron microscopy (SEM) micrographs were anonymized and coded with randomized alphanumeric identifiers prior to quantitative analysis.

Each digital micrograph was imported into ImageJ software (version 1.54, National Institutes of Health, Bethesda, MA) and cropped to a standardized fixed dimension of 263,760 pixels to evaluate the percentage of clean dentin surface area relative to the total canal surface area. All measurements were conducted by a single trained investigator and repeated after a two-week interval to assess intra-examiner reliability. The intraclass correlation coefficient (ICC) was calculated, yielding a value of 0.94, which demonstrated excellent measurement reproducibility.

Statistical analysis

Statistical analysis was conducted using Statistical Product and Service Solutions (SPSS, version 21.0; IBM SPSS Statistics for Windows, Armonk, NY). A one-way analysis of variance (ANOVA) was performed to evaluate the differences between the four groups within each dental region. For intra-group comparisons across the three regions (apical, middle, and coronal), a repeated measures ANOVA was used. The normality of the data distribution was assessed using the Shapiro-Wilk test. To account for minor departures from normal distribution observed in two sub-groups (Group I coronal and Group IV apical), a non-parametric sensitivity analysis using the Kruskal-Wallis test was also performed across all three dental regions. When any ANOVA yielded statistically significant results (p < 0.05), a Bonferroni post-hoc test was applied for multiple pairwise comparisons. A p-value of less than 0.05 was considered statistically significant.

## Results

The result from the ImageJ software was expressed in the form of a percentage between the cleaned area and total area in all three regions of all the groups. Values are expressed as mean and standard deviation (SD) (Table 1).

**Table 1:** Statistical analysis of group measurements across dental regions Means followed by different superscript letters (a, b, c, d) are significantly different from one another (p < 0.0001) according to a post-hoc test. For all regions (apical, middle, and coronal), the overall significance level was established at p < 0.001. Superscripts represent the experimental groups as follows: a = Group I (conventional syringe irrigation); b = Group II (sonic irrigation); c = Group III (passive ultrasonic irrigation); d = Group IV (laser diode irrigation).

Group	Apical		Middle		Coronal	
	Mean	SD	Mean	SD	Mean	SD
Group I	45.1^a^	2.43	47.25^a^	1.92	51.4^a^	1.98
Group II	54.2^b^	2.26	59.4^b^	1.67	61.05^b^	1.76
Group III	64.45^c^	3.66	65.05^c^	1.64	67.75^c^	1.71
Group IV	72.3^d^	0.98	74.15^d^	1.18	75.3^d^	1.49
P value	0.0001		0.0001		0.0001	

The distribution of data normality across the regions was verified using the Shapiro-Wilk test (Table 2). The table reveals that the majority of the data groups across the apical, middle, and coronal regions follow a normal distribution (p> 0.05). However, the coronal section of Group I (p = 0.0098) and the apical section of Group IV (p= 0.0105) were not normally distributed.

**Table 2:** Normality test using the Shapiro-Wilk normality test

Group	Shapiro-Wilk normality test (P value)		
	Apical	Middle	Coronal
Group I	0.6816	0.3036	0.0098
Group II	0.496	0.5154	0.1824
Group III	0.1847	0.0805	0.0727
Group IV	0.0105	0.0699	0.211

Furthermore, a highly significant variance among all four groups across the apical, middle, and coronal regions was confirmed via intergroup analysis (Table 3). Statistical analysis revealed highly significant differences among all four groups across the apical, middle, and coronal regions (p < 0.0001). Group IV consistently exhibited the highest mean values across all locations, followed by Group III and Group II, while Group I recorded the lowest values. Post-hoc pair-wise comparisons confirmed that every group was statistically distinct from one another at all three levels (p < 0.0001 for all comparisons). Within each specific region, there was a progressive and significant increase in measurements from Group I through Group IV, indicating that the experimental variables uniquely and significantly impacted each group's results. A non-parametric sensitivity analysis using the Kruskal-Wallis test confirmed these findings (p < 0.0001), demonstrating identical significant differences and performance ranking among the four groups across all root canal regions.

**Table 3:** Bonferroni post-hoc test for intergroup analysis (unique pairwise comparisons)

(I) Group	(J) Group	Apical (p-value)	Middle (p-value)	Coronal (p-value)
Group I	Group II	0.0001	0.0001	0.0001
Group I	Group III	0.0001	0.0001	0.0001
Group I	Group IV	0.0001	0.0001	0.0001
Group II	Group III	0.0001	0.0001	0.0001
Group II	Group IV	0.0001	0.0001	0.0001
Group III	Group IV	0.0001	0.0001	0.0001

**Figure 1:**
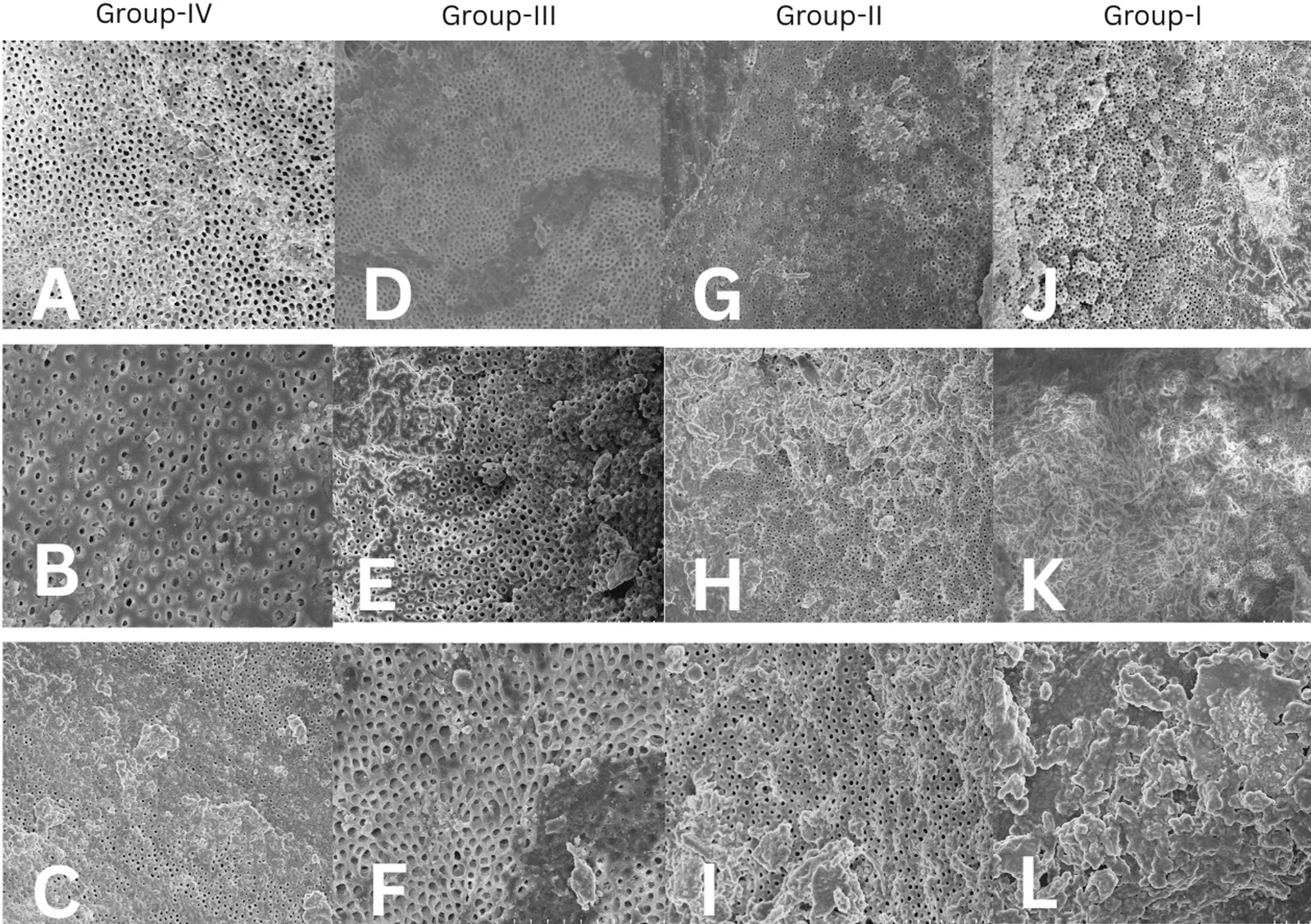
Representative scanning electron micrographs (x10,000 magnification) showing residual bioceramic sealer distribution across root canal regions (coronal, middle, and apical thirds) (A-C) Group IV (laser diode); (D-F) Group III (passive ultrasonic); (G-I) Group II (sonic); (J-L) Group I (conventional syringe irrigation)

Representative scanning electron microscopic images demonstrating the comparative residual sealer distribution across the coronal, middle, and apical thirds for all four groups are displayed in Figure 1.

## Discussion

The success of retreatment initially depends on proper removal of obturating material (gutta-percha and sealer), which remains a genuine clinical difficulty [15]. During retreatment, the removal of obturation material placed via warm compaction techniques presents a more significant challenge than other methods [16].

Materials that are used to fill the canal need to be removed during retreatment and, nowadays, newer materials, such as a BC sealer [17], which is thought to be the best for forming apatite crystal-like structure, have a stronger bond and faster setting time, but the main difficulty of using BC sealer is retrieval from the canal [3]. During the removal process, these sealers leave residual materials that act as a substrate for persistent bacterial colonization, limiting successful treatment outcomes and ultimately leading to failure [18].

Obturation material removal is done with hand files, which is time-consuming and leaves more residual material than the rotary system [1]. Although rotary systems, such as Protaper Universal, show better removal of material, they do not achieve complete removal [19]. To achieve better antibacterial action and more removal of obturating material, an adjunct irrigation activation technique is used along with the Protaper Universal rotary system [20].

In the current study, the retreatability of a BC sealer used in combination with a warm vertical compaction technique was evaluated in single-rooted premolars. Surface cleanliness was compared following the use of a 980-nm laser diode, conventional syringe irrigation, sonic activation, and passive ultrasonic activation.

Following the mechanical removal of gutta-percha, chemical disinfection and physical debridement are necessary [21]. Conventional syringe irrigation delivers an irrigant flow extending only 1-1.5 mm beyond the needle tip, which represents a major limitation of the technique [6]. The clinical effectiveness of an irrigant depends heavily on its route of delivery and fluid dynamics within complex root anatomy [22]. To optimize these dynamics, various activation protocols have been developed, including sonic, ultrasonic, and laser activation [23].

Sonic and ultrasonic activation methods work on the principles of acoustic streaming and cavitation, though they operate at completely different frequencies. Sonic devices function at approximately 5,000-6,000 Hz, whereas ultrasonic systems operate at around 30,000-45,000 Hz [8]. This difference in operating frequency explains why ultrasonic activation consistently outperforms sonic activation in clinical simulations [24].

Passive ultrasonic irrigation relies on acoustic streaming generated via oscillating metallic files, which is frequently dampened by physical file-to-wall contact inside the canal. While laser-activated irrigation (LAI) is well-documented with mid-infrared Erbium lasers (Er:YAG/Er,Cr:YSGG), direct comparative literature specifically evaluating 980-nm diode laser activation against passive ultrasonic irrigation for BC sealer retreatment remains scarce - providing the core rationale for this investigation. Near-infrared 980-nm diode lasers deliver concentrated thermal energy into the aqueous irrigant, rapidly reaching the energy threshold required to induce the formation, rapid growth, and violent implosion (cavitation) of vapor bubbles. The implosion of these micro-bubbles generates transient shockwaves, high-velocity fluid microstreaming, and severe wall shear stresses that effectively detach and flush out resilient, micro-anchored BC sealer particles from dentinal surfaces [24,25]. In contrast to passive ultrasonic irrigation, the flexible, contactless 200-µm fiber optic tip of the 980-nm diode laser eliminates mechanical dampening, allowing unrestricted photothermal cavitation and hydrodynamic streaming to extend deep into the apical third.

In this study, laser diode irrigation (Group IV) achieved statistically superior canal cleanliness (72.3%-75.3%) across all root canal thirds compared to passive ultrasonic irrigation (64.45%-67.75%), sonic activation (54.2%-61.05%), and conventional syringe irrigation (45.1%-51.4%) (p < 0.0001), leading to the rejection of the null hypothesis.

In the apical third, the cleaning efficiency of the laser group was superior, followed by passive ultrasonic irrigation, sonic activation, and conventional syringe irrigation. These findings agree with those of Abaza et al. [11] and Elkhodary et al. [12], who concluded that laser diode activation was superior to both passive ultrasonic and conventional syringe irrigation in removing debris from apical sections. Furthermore, the superiority of the laser and ultrasonic groups over sonic and conventional syringe methods matches the patterns observed by Goker et al. [26]. The finding that passive ultrasonic irrigation provides better apical cleanliness than sonic or conventional syringe irrigation also mirrors the results reported by Grischke et al. [27].

The current study also showed that sonic irrigation achieves superior cleanliness in the coronal third compared to the apical third, which mimics the findings of Goker Kamali et al.'s study [26]. The coronal third consistently demonstrated excellent results regarding sealer removal across all groups. This stands in contrast to the apical third, where physical constraints and complex anatomy present a severe challenge to fluid circulation. This regional variance in cleanliness was evident across the laser diode, passive ultrasonic, and conventional syringe irrigation groups, matching observations by Ekim et al. [28], that the coronal third is invariably cleaner than its apical counterpart.

A primary limitation of this study is its in vitro design using extracted straight, single-rooted premolars, which standardized baseline parameters but failed to replicate complex root canal anatomies, severe curvatures, surrounding periodontal tissue responses, dynamic intra-canal fluid pressure, or temperature shifts occurring in vivo. Furthermore, the evaluation was restricted to a single BC sealer and warm vertical compaction technique. Finally, canal cleanliness was quantified using two-dimensional SEM micrographs rather than non-destructive, three-dimensional micro-computed tomography (micro-CT) volumetric analysis.

## Conclusions

Within the limitations of this in vitro SEM study, it can be concluded that none of the evaluated irrigation activation techniques achieved complete removal of the BC sealer from the root canal system, highlighting the persistent challenge of endodontic retreatment. However, significant differences were observed among the tested activation systems: diode laser activation (980 nm) demonstrated the highest efficacy in removing BC sealer across all regions of the root canal, followed sequentially by passive ultrasonic irrigation, sonic activation, and conventional syringe irrigation. The superior performance of the diode laser is attributed to its advanced cavitation effects and enhanced fluid dynamics, which improve debridement efficiency within complex canal anatomies. Passive ultrasonic irrigation also showed substantial effectiveness driven by acoustic streaming and cavitation, whereas sonic activation provided only moderate improvement over conventional methods. Conventional syringe irrigation exhibited the least effectiveness, primarily due to limited irrigant penetration and a lack of fluid activation dynamics. Additionally, all groups demonstrated significantly greater cleanliness in the coronal third compared to the apical third, confirming that anatomical constraints and limited accessibility continue to hinder effective debridement in the apical region. Therefore, while irrigant activation significantly enhances BC sealer removal during retreatment, diode laser activation emerged as the most effective adjunctive technique among those evaluated. Further in vivo investigations and studies utilizing complex root canal geometries are warranted to validate these findings and optimize their clinical applicability.
